# Receptor modulators associated with the hypothalamus -pituitary-thyroid axis

**DOI:** 10.3389/fphar.2023.1291856

**Published:** 2023-12-04

**Authors:** Xianbin Cheng, Hong Zhang, Shanshan Guan, Qi Zhao, Yaming Shan

**Affiliations:** ^1^ National Engineering Laboratory for AIDS Vaccine, School of Life Sciences, Jilin University, Changchun, China; ^2^ Department of Thyroid Surgery, The Second Hospital of Jilin University, Changchun, China; ^3^ Postdoctoral Research Workstation, Changchun Gangheng Electronics Company Limited, Changchun, China; ^4^ College of Biology and Food Engineering, Jilin Engineering Normal University, Changchun, China; ^5^ Cancer Centre, Faculty of Health Sciences, University of Macau, Taipa, China; ^6^ Key Laboratory for Molecular Enzymology and Engineering, The Ministry of Education, School of Life Sciences, Jilin University, Changchun, China

**Keywords:** HPT, receptor, modulator, agonist, antagonist

## Abstract

The hypothalamus-pituitary-thyroid (HPT) axis maintains normal metabolic balance and homeostasis in the human body through positive and negative feedback regulation. Its main regulatory mode is the secretion of thyrotropin (TSH), thyroid hormones (TH), and thyrotropin-releasing hormone (TRH). By binding to their corresponding receptors, they are involved in the development and progression of several systemic diseases, including digestive, cardiovascular, and central nervous system diseases. The HPT axis-related receptors include thyrotropin receptor (TSHR), thyroid hormone receptor (TR), and thyrotropin-releasing hormone receptor (TRHR). Recently, research on regulators has become popular in the field of biology. Several HPT axis-related receptor modulators have been used for clinical treatment. This study reviews the developments and recent findings on HPT axis-related receptor modulators. This will provide a theoretical basis for the development and utilisation of new modulators of the HPT axis receptors.

## 1 Introduction

The hypothalamus-pituitary-thyroid (HPT) axis is responsible for the release of thyroid hormones (TH) and participates in the occurrence and development of various diseases. (Feldt-Rasmussen et al., 2021). After the thyrotropin-releasing hormone (TRH) secreted by the hypothalamus binds with the thyrotropin-releasing hormone receptor (TRHR) on the cell membrane of pituitary thyrotropin, it activates the intracellular signalling pathway of the thyrotropin cells and promotes the secretion and synthesis of thyrotropin (TSH). (Lazcano et al., 2021). TSH released from the pituitary gland is bound with thyrotropin receptor (TSHR) on the membrane of thyroid follicular epithelial cells through blood circulation to promote thyroid release and synthesis After being released into the blood, TH are transported to various tissues of the body in a free state or in the form of bound plasma protein. (Ortiga-Carvalho et al., 2016). TH binds to the thyroid hormone receptor (TR) to produce corresponding biological effects. TH functions in almost all organs and tissues of the body, regulates material and energy metabolism, and affects individual growth and development (Cioffi et al., 2022).

TH released by the HPT axis is an important human hormone and master regulator of cell growth, development, and maintenance of tissue homeostasis. TH can regulate adipocyte differentiation or induce the hepatic synthesis of multiple fat-metabolising enzymes. TH can accelerate both the synthesis and decomposition of cholesterol, but decomposition is greater than synthesis (Mullur et al., 2014). TH improves oxygen consumption and thermogenesis in tissues such as fat, skeletal muscle, cardiac muscle, liver, and kidney. TH also potentiates protein breakdown and promotes sugar absorption, utilisation, and glycogen synthesis. Additionally, TH can accelerate gastrointestinal motility and gastric emptying and reduce intestinal absorption (Nakazawa et al., 2021). Foetuses with congenital thyroid dysgenesis show skeletal growth arrest after birth because of TH (Wassner, 2018). Meanwhile, even small alterations in blood TH levels can lead to changes in a range of TH related phenotypes, such as altered body weight, adiposity, bone mineral density, and heart rate (Wolf et al., 2017).

**FIGURE 1:**
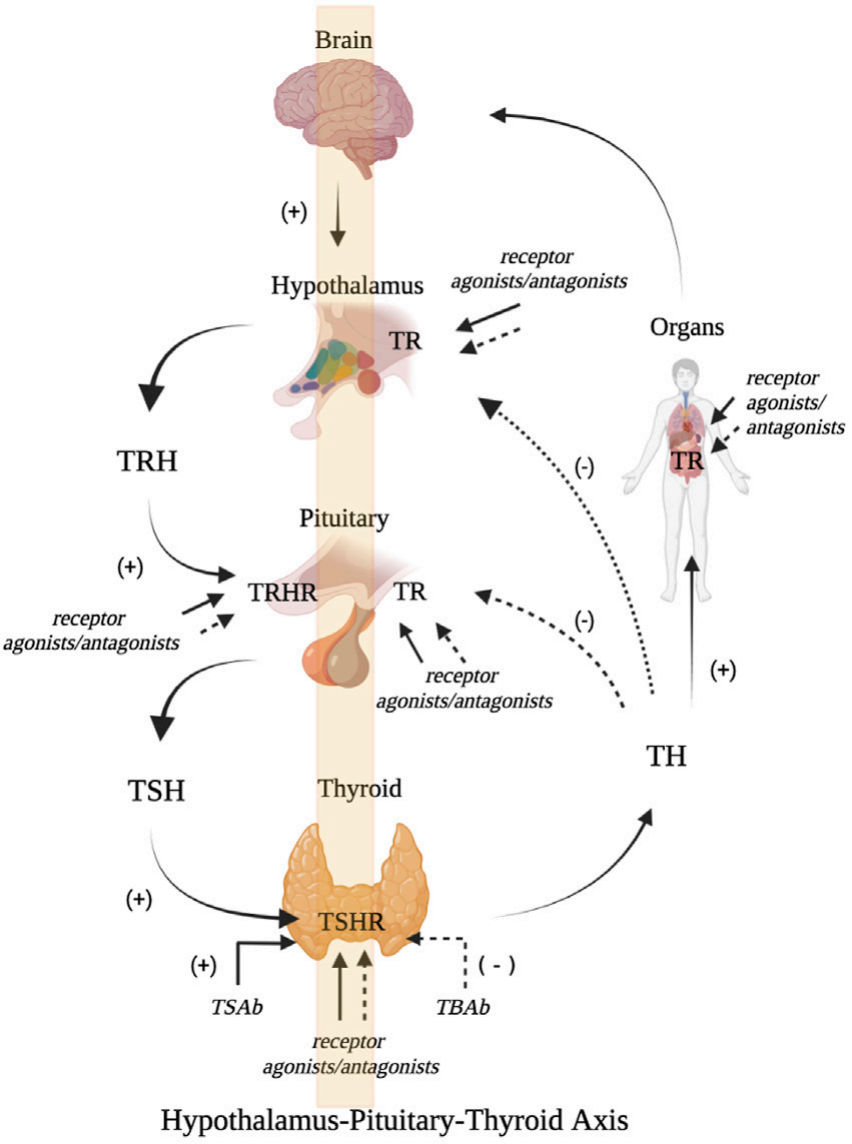
Schematic diagram of HPT axis related receptor regulation **(A)**. The hypothalamus secretes TRH which binds with TRHR to stimulate the release of pituitary TSH. TSH further promotes the generation and release of TH by activating TSHR. TH can bind with TR, which can not only promote growth and development, but also maintain internal environment disorder. It can also inhibit the secretion of TRH and TSH through negative feedback **(B)**. Receptor modulators can act on TRHR, TSHR and TR, and stimulate or antagonize the biological activities of TRH, TSH and TR (Figure is produced by https://biorender.com/).

Negative feedback regulation is crucial in the regulation mechanism related to the HPT axis. TRH neurones in the hypophysotropic zone of the paraventricular hypothalamic nucleus (PVN) are the main sites for TRH secretion. Studies have identified three negative thyroid hormone response elements (TREs) in the promoter region of human TRH. TR can bind to TREs as monomers, homodimers, and heterodimers, which in turn mediates the negative feedback regulation of TRH target genes by TH (Weiner et al., 2021). The regulation of the HPT axis not only correlates with circulating TH levels but also requires the joint participation of multiple molecules. This allows circulating TH to enter the central nervous system (CNS) and exert a regulatory effect. TH exerts its biological effects via nuclear receptors. TH must exert its regulatory role within the nuclei of TRH neurones by crossing the blood-brain and blood–CSF barriers through TH transporters. After entering cells, the interconversion of different active forms of TH are regulated by intracellular (tanycyte) deiodinases (Dios). After entering the nucleus of TRH neurones, similar to most nuclear receptors, the transcriptional regulation of genes by TH are also dependent on the participation of TR and cofactors. In addition, pyroglutamyl peptidase II (PPII) expressed by cells can degrade TRH. Thus, the TRH levels that reach the pituitary portal vein in different thyroid functional states are affected. Thyroid function and blood TH levels are primarily regulated by TRH and TSH. TH represses the pituitary TRHR gene, TSH β gene expression, and TSH secretion via negative feedback regulation. (Costa-e-Sousa and Hollenberg, 2012). But the negative feedback regulatory point of TH are controlled by TRH levels. It follows that TSH, TH, and TRH are important in the HPT axis and play important roles through their corresponding receptors: TSHR, TR, and TRHR. (Figure1).

As a central link receptor of the HPT, TSHR is a member of the G-protein-coupled receptor (GPCRs) superfamily, the main antigen of autoimmune thyroid diseases, and can be used as a drug target for Graves’ disease (GD). TSHR is also an important protein that regulates the occurrence and development of thyroid cancer and is therefore a potential target for the diagnosis and treatment of thyroid cancer. Research has shown that TSHR regulators have an interventional effect on experimental models of thyroid diseases such as GD and thyroid cancer (Ladenson et al., 2010a). Compared with TSHR, TR, a member of the nuclear receptor family, is responsible for regulating cell differentiation, development, metabolism, and physiological functions in most tissues (Davies and Latif, 2015a). TR have become targets for many diseases. Several TR regulators have been synthesised for targeted therapies. Ladenson et al (Ladenson et al., 2010b) published clinical trial results of the TR regulator eprotirome to reduce blood lipids in the New England Journal of Medicine, indicating the possibility of a TR regulator entering the clinic. In addition, TRH, the most important regulator of HPT, exerts biological effects when bound with TRHR. Kato et al (Kato et al., 2009) reported that the muscular strength of patients with type III spinal muscular atrophy significantly improved after oral administration of taltireline hydrate, a TRH receptor modulator. It has been approved for the treatment of spinal cerebellar degeneration in Japan (Khomane et al., 2011a).

Concomitant with the progress in global science and technology, the development and synthesis of various receptor modulators have gradually improved. Receptors and their corresponding regulators have become a popular topic in modern biological research. Many previous studies have reported modulators of HPT axis-related receptors.

Receptor modulators are currently used in clinical studies. However, there are still relatively few receptor modulators have been used to treat HPT axis-related diseases. Taltirelin is already used in clinical practice, but it can also cause adverse gastrointestinal reactions, such as nausea and vomiting. For the treatment of autoimmune thyroid disease (AITD), antithyroid drugs such as thiamazole remain the main treatment schemes for GD patients. Oral thyroid hormones are first-line clinical treatments for patients with hypothyroidism. Currently, there is an urgent need to develop a receptor modulator with clear therapeutic effects and minimal side effects for the treatment of HPT axis-related diseases.

In this review, we obtained relevant research by searching online databases. And we describe receptor modulators targeting TSHR, TR, and TRHR and discuss the mechanism of action of these modulators and related receptors. The potential clinical applications of HPT axis-related receptor modulators in diseases were reviewed to provide a theoretical basis for the development of new modulators.

## 2 Thyrotropin receptor

TSHR belong to the family receptor (GPCRs family and have a molecular weight of 84.5Ku. TSHR is located on chromosome 14q32 and is approximately 60 kb long with 10 exons and 9 introns. The encoded TSHR protein consists of 764 amino acid residues and is divided into three parts: extramembrane, transmembrane, and intramembrane regions. (Núñez Miguel et al., 2022). Exons one to nine encode the extramembranous region at the N-terminus of TSHR, whereas exon 10 encodes the transmembrane and intramembrane regions. There are 418 amino acid residues in the outer membrane region that are hydrophilic and contain an N-terminus, five glycosylation sites, and two immunogenic peptides. There are 264 amino acid residues in the transmembrane region, which are hydrophobic and have seven transmembrane segments, and the outer and inner membrane regions form three loops each. The intramembrane region contains 82 amino acids.

The extramembrane region of TSHR is an important site for binding to TSH and TSH receptor antibodies (TRAb). TSH has multiple discrete binding sites in its extra-membrane region. TRAb also has a binding epitope in the extramembrane region, but both bind to TSHR with varying affinities. TRAb also has a binding epitope in the extramembrane region, and its affinity of TRAb binding to TSHR is different from that for TSH. The TSH- and TRAb-binding sites on TSHR were distributed in the outer membrane region, with little overlap in the segment from amino acids 1 to 260, but in the segment from amino acids 261 to 418. The two different TRAb act on different TSHR-binding epitopes and produce distinct clinical presentations. Studies have shown that the binding of TSHR to its ligands is related to the configuration and glycosylation of the outer membrane region. When this region is folded or glycosylated, it enhances the immunogenicity of TSHR and generates more antibody-binding epitopes. (McLachlan and Rapoport, 2017). The transmembrane region of TSHR affects information transmission, maintenance of its three-dimensional structure, and biological activity, whereas the intramembrane region is coupled to G proteins and exerts its effects.

Current research has found that treatment with TSHR as a target through TSHR modulators has a positive effect on the treatment of autoimmune thyroid diseases and thyroid cancer. (Rowe et al., 2017). We provide a summary of TSHR regulators. Currently, TSHR receptor modulators mainly include two types: TSHR monoclonal antibodies and small-molecule ligands.

### 2.1 TSHR monoclonal antibody

**FIGURE 2:**
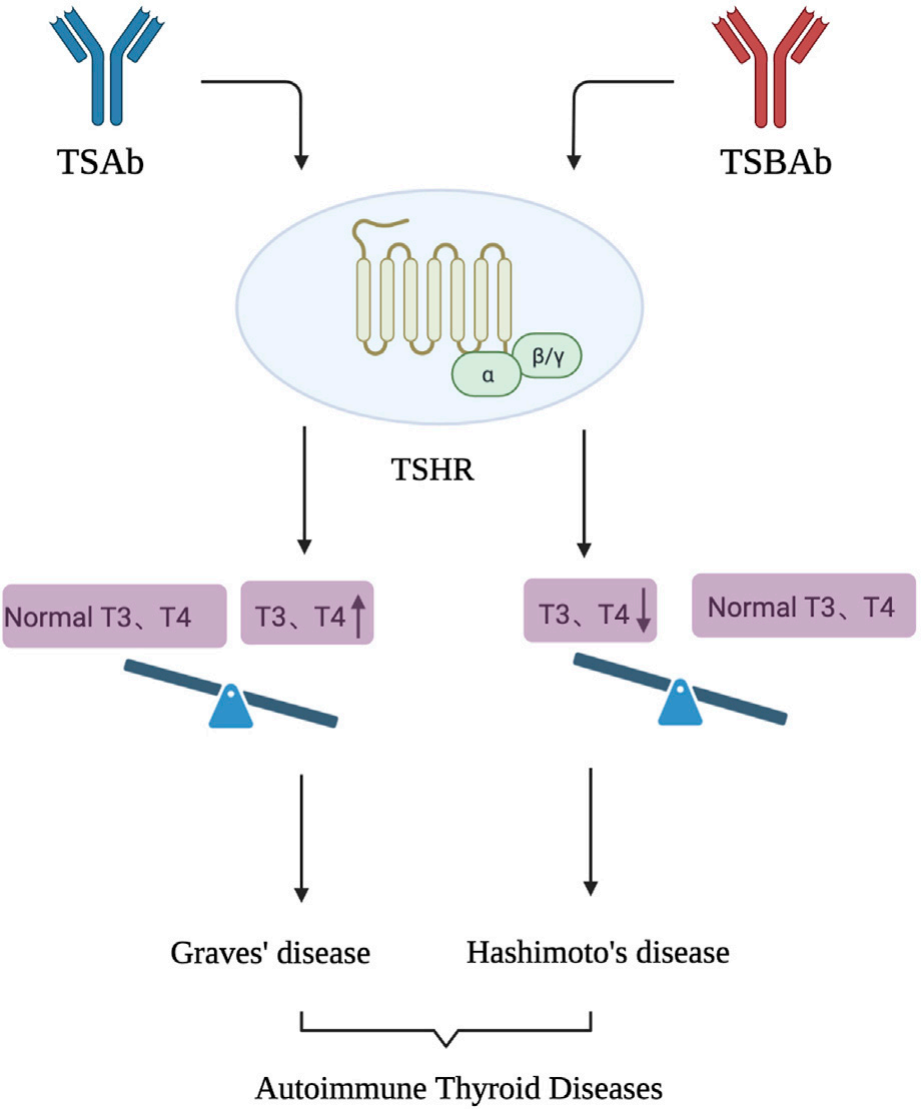
TSAb and TSBAb, as antibodies to TSHR, act on TSHR, leading to the occurrence of AITD. AITD is an organ specific autoimmune disease that mainly includes GD and Hashimoto’s thyroiditis. TSAb can activate TSHR and produce biological effects similar to TSH, causing hyperthyroidism. After binding with TSHR, TSBAb blocks the binding of TSH to the receptor and inhibits the biological effect of TSH, causing hypothyroidism.

AITD is an organ-specific autoimmune disease that includes GD and Hashimoto’s thyroiditis. (Bogusławska et al., 2022). Its onset is closely related to that of the TRAb. TRAb is a heterogeneous group of antibodies against TSHR and a regulator of TSHR that acts on TSHR on the thyroid membrane surface to produce different biological effects. (Lee et al., 2022) (Figure 2).

TRAb are divided into the following categories according to their functions. (Morshed et al., 2018). 1) TSH-stimulating antibody (TSAb) can activate TSHR and produce biological effects similar to those of TSH, causing hyperthyroidism. 2) TSH stimulation-blocking antibody (TSBAb): After binding to TSHR, it blocks the binding of TSH to its receptor and inhibits the biological effect of TSH, causing hypothyroidism. 3) Neutral TSH receptor antibodies often exist in the serum of patients with GD. It cannot activate the G protein-dependent signal transduction pathway, leading to the production of cyclic adenosine monophosphate (cAMP); however, it can induce cell apoptosis through a variety of stress signalling pathways.

TSAb and TSBAb have important clinical significance in the diagnosis, differential diagnosis, evaluation of efficacy, determination of drug withdrawal time, and monitoring of high-risk groups for AITD and other diseases. (Kahaly et al., 2020). The TRAb epitope, which is a discontinuous site, mainly exists in the outer membrane of TSHR. Different TRAb have been used to identify different TSHR epitopes. The binding site of TSAb is mainly located at the (N terminal) of the extracellular domain of TSHR, whereas the binding site of TSBAb is concentrated at the carboxyl terminal, and the binding site of the neutral antibody is the hinge region of the extracellular domain of TSHR. (Morshed et al., 2019). TRAb has low content and purity in the human body, and it is difficult to separate different functional antibodies, which limits its direct extraction and purification from patients with GD. Therefore, researchers have begun to prepare a variety of monoclonal antibodies against TSHR, which have significant advantages such as high purity, strong specificity, high titre, and few cross-reactions. As regulators of TSHR, monoclonal antibodies against TSHR have shown broad prospects in the pathogenesis, detection, and development of immunotherapy methods for TRAb-related diseases.

#### 2.1.1 M22 and K1-18

In 2003, Sanders et al. (Sanders et al., 2003) reported that the first humanized TSHR monoclonal antibody, M22, was prepared from peripheral blood B lymphocytes of a 19-year-old male patient with Gd and type I diabetes. The antibody and Fab fragment have high affinity for TSHR, which antagonises the binding of TSH and TSHR and stimulates TSHR. It can be widely used as a ligand to replace bovine TSH for the detection of TRAb, with good sensitivity and specificity. Evans et al. (Evans et al., 2010) isolated the thyroid-stimulating autoantibody K1-18 from the peripheral blood lymphocytes of a woman with an 8-year history of AITD, which has the potential to replace recombinant TSH. Both M22 and K1-18 showed high binding affinities for TSHR. M22 and K1-18 inhibited the binding of ^125^I-TSH and TSHR in a dose-dependent manner, inhibiting 80% and 72%, respectively, at 100 ng/mL, and 22% and 21%, respectively, at 10 ng/mL. Both M22 and K1-18 effectively inhibited the mutual binding and the binding of patient serum TRAb and TSHR. (Rees Smith et al., 2009; Evans et al., 2010). In addition, both antibodies are complete agonists of TSHR and have the same maximum stimulatory effect on the production of cAMP as TSH.

##### 2.1.2 5C9 and K1-70

Evans et al (Evans et al., 2010) isolated the thyroid-stimulating blocking monoclonal antibody K1-70 from the peripheral blood lymphocytes of the same patient as K1-18. This is the first study to provide direct evidence of the simultaneous existence of TRAb with different activities in patients. In 2022, Duan et al (Duan et al., 2022) first identified the key amino acid residues specifically recognised by TSH and TSHR. Through the comparison, analysis and verification of the receptor structures in the inactivated and activated states, a universal model of hormone activated glycoprotein hormone receptor, namely, “Push and Pull” model, is proposed. Through the structural analysis of the activated antibody M22 and TSHR, it was revealed that the antibody activated TSHR mainly through “push”. Structural analysis of the blocking monoclonal antibodies K1-70 and TSHR revealed that K1-70 can stably bind to the surface of the deactivated TSHR extracellular domain (ECD). When TSH binds to the inactivated receptor ECD, it will produce steric hindrance with the cell membrane, thus promoting the upward deflection of the receptor ECD, that is, the role of “pull”. This study revealed the molecular mechanism underlying the interaction between K1-70 and TSHR.

The research team isolated the thyroid-stimulating monoclonal autoantibody 5C9 from the peripheral blood lymphocytes of a 27-year-old patient with postpartum hypothyroidism. (Furmaniak et al., 2012). K1-70 and 5C9 also had similarly high binding affinities for TSHR. (Nakatake et al., 2006). K1-70 and 5C9 inhibited the combination of ^125^I-TSH and TSHR in a dose-dependent manner, and K1-70 was more effective than 5C9. K1-70 and 5C9 are effective inhibitors of TSH production in CHO-TSHR cells. (Rees Smith et al., 2009). *In vitro*, K1-70 and 5C9 inhibited TSH-induced cAMP production by 61.6%, 62.3%, 86.9%, and 81.6% at 50 and 100 ng/mL, respectively. The two autoantibodies did not show any stimulatory activity within a certain concentration range, indicating that they two monoclonal antibodies were pure antagonists. Thus, K1-70 can be used to treat thyroid crises *in vivo* to shorten the course of GD. 5C9 inhibits the constitutive activity of TSHR and controls TSHR-related hyperthyroidism and thyroid cancer. In addition, blocking mAbs cannot penetrate the placenta, control the symptoms of hyperthyroidism in pregnant women, and have good application prospects. (Lee et al., 2011).

### 2.2 TSHR small molecule ligand

Small molecules have the advantages of easy and rapid passage through the cell membrane, convenient and safe oral administration, and inexpensive synthesis. Small-molecule ligand regulators targeting TSHR have great potential for treating TSHR-related diseases. (Neumann and Gershengorn, 2011). Small molecule ligand regulators of TSHR include agonists, inverse agonists, and antagonists (Table 1).

**TABLE 1:** Some small molecule ligands of thyrotropin receptor.

Year	Researchers	Name	2D structure	3D status	Action
2006	Jäschke et al., 2006	Org41841	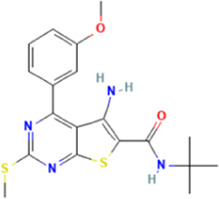	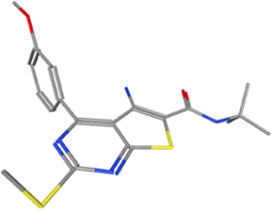	Agonist
2009	Neumann et al., 2009	NCGC00161870	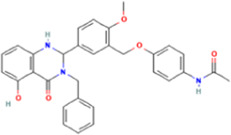	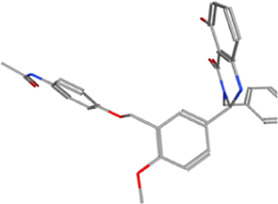	Agonist
		(C2/ML-109)			
2010	Neumann et al., 2010	NCGC00161856	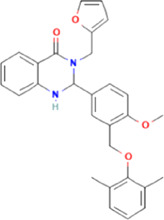	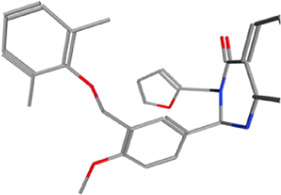	Antagonist
2011	Neumann et al., 2011	NCGC00229600	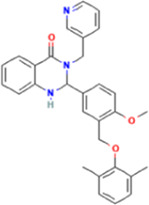	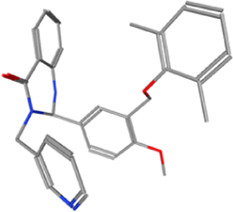	Inverse agonist
2014	Neumann et al., 2014	NCGC00242364 (ML-224/ANTAG-3)	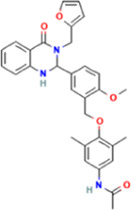	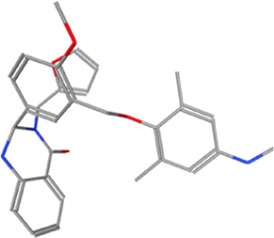	Antagonist
2015	Latif et al., 2015	MS437	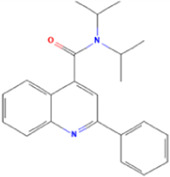	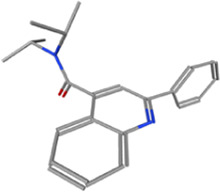	Agonist
2015	Latif et al., 2015	MS438	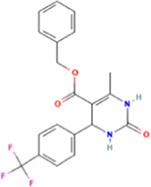	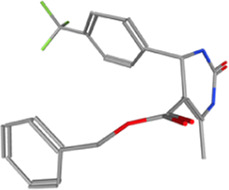	Agonist
2019	Marcinkowski et al., 2019	S37a	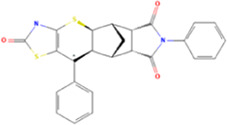	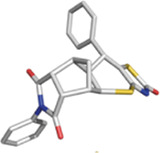	Antagonist
2020	Latif et al., 2020	MSq1	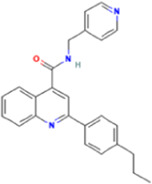	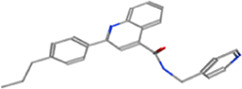	Agonist

The results show that the small-molecule ligand of TSHR binds with the amino acids of seven transmembrane helices to exert a biological regulatory effect. (Haas et al., 2011). Small molecule ligands can activate or block these binding sites through conformational modulation. The rationale for this is that the receptor has an allosteric centre in addition to an active centre. These centres can bind to ligands and change the conformation of the enzyme, thereby affecting the function of the receptor (Davies and Latif, 2015b). A possible mechanism for the inhibition is that small molecule ligands stabilise the ionic lock in the transmembrane domain structure. An ionic lock is a polar interaction between different amino acids to form a transmembrane helix. When TSHR antibodies or TSH bind to the receptor ectodomain, the ionic lock restricts the polar contacts of parts of the transmembrane helices, leaving no room for movement, which in turn prevents G protein coupling with activation. This prevents signalling by blocking the essential structure of TSH. This antagonist maintains the ionic lock in the transmembrane region, which tends to stabilise. In contrast, small-molecule agonists of TSHR may activate the receptor by disrupting the structural stabilisation of the ionic lock as well as polar interactions between the helices (Audet and Bouvier, 2012).

#### 2.2.1 NCGC00161870

Neumann et al (Neumann et al., 2009) used quantitative high-throughput screening to identify the small-molecule TSHR agonist NCGC00161870. The other names are C2 and ML-109. This agonist is highly selective for human TSHR compared to other glycoprotein hormone receptors. In primary cultures of human thyroid cells, both TSH and its agonists increased the mRNA levels of thyroglobulin, thyroperoxidase, sodium iodide transporter, type 2 deiodinase, and type 2 deiodinase. Additionally, oral agonists stimulate thyroid function in mice, resulting in increased serum thyroxine and thyroid radioiodine uptake.

Further studies were conducted to elucidate the mechanism of action of NCGC00161870. They found that NCGC00161870 is a racemic mixture containing equal amounts of the enantiomers E1 and E2. E1 and E2 bind differently to a homology model of the TSHR transmembrane domain, in which E2 has a lower binding energy than E1 and is therefore predicted to be more potent than E1. In HEK293 cells expressing human TSHR, E1 and E2 were more potent than E1 alone in stimulating cAMP production. In primary cultured human thyrocytes, E1 and E2 stimulated thyroid peroxidase mRNA levels were 55 and 137fold higher than basal levels, respectively, and sodium iodide transporter mRNA levels were 4 and 121fold higher, respectively. In mice, E2 stimulated a 2.8fold increase in radioiodine uptake, whereas E1 had no such effect. Oral administration of E2 for 5 days increased serum T4 levels compared with recombinant human TSH (rhTSH). Therefore, E2 is more effective than E1 in stimulating thyroid function and is as effective as rhTSH *in vivo*. (Neumann et al., 2016). NCGC00161870 binds to TSHR mainly through E2, with corresponding biological effects. Studies have shown that NCGC00161870 should be further investigated as an oral agent for patients with thyroid cancer.

Duan et al (Duan et al., 2022) systematically studied the structure of TSHR with the small-molecule agonist NCGC00161870and revealed the mechanism by which NCGC00161870 induces receptor activation for the first time. The interaction between NCGC00161870 and the TSHR was almost completely hydrophobic. Mutation experiments of amino acids at the transmembrane region binding site revealed that mutations in M572A, V586A, I640A, A644F, I648A, and L662A in the transmembrane region significantly reduced the ability of NCGC00161870 to activate TSHR. These amino acid residues interact with NCGC00161870. These results provide a clear basis for the discovery of small-molecule drugs for the treatment of autoimmune thyroid diseases caused by abnormal TSHR function.

#### 2.2.2 MS437 and MS438

Latif et al (Latif et al., 2015) screened two small-molecule regulators of TSHR, MS437 and MS438, using a high-throughput screening system. The ability of these small-molecule agonists to bind to the transmembrane domain of the receptor and initiate signal transduction is exemplified by the activation of a chimeric receptor consisting of the outer domain of LHR and the transmembrane domain of TSHR. It was found by luciferase reporter that MS437 and MS438 exhibited similar results to TSH for efficient activation of the site of Gsα、Gαq and Gα12 on TSHR. MS437 and MS438 also upregulated the expression of thyroglobulin (TG), sodium iodide transporter (NIS) and TSHR. The pharmacokinetic analysis of MS437 and MS438 suggested their potential as pharmacotherapeutic agents. Intraperitoneal injection of MS437 and MS438 into normal female mice resulted in a significant increase in serum thyroxine levels, which was maintained by repeated treatments. Therefore, MS437 and MS438 could be used as TSHR agonists for further development.

#### 2.2.3 NCGC00229600

NCGC00229600 is an inverse TSHR agonist. Traditionally, regulators are classified as antagonists and agonists. Those that can produce biological effects are called agonists and those that cannot produce biological effects are called antagonists. Recent studies have found that some antagonists can also produce biological effects; these regulators are called inverse agonists. GD is caused by persistent, unregulated stimulation of thyroid cells by activated TSAb. TSHR small-molecule antagonists that inhibit serum-stimulated receptor signalling in patients with GD NCGC00229600 inhibited TSH-stimulated cAMP generation. Neumann et al. (Neumann et al., 2011) found that NCGC00229600 inhibited cAMP production in the sera of 30 GD patients. NCGC00229600 is not only used to treat GD but can also reduce the symptoms of Graves’ ophthalmopathy. NCGC00229600 inhibited the production of cAMP, pAkt, and HA in undifferentiated orbital fibroblasts and can be used to treat GD ophthalmopathy. (Turcu et al., 2013).

##### 2.2.4 S37a

Graves’ orbitopathy (GO) can occur in patients with Graves’ disease due to pathogenic activation of TSHR in retro orbital fibroblasts. Marcinkowski et al (Marcinkowski et al., 2019) identified TSHR inhibitors by high-throughput screening. Thus, S37a was screened as a TSHR antagonist. It inhibits TSH induced cAMP accumulation in HEK 293 cells expressing TSHR. The unique rigid bending shape of S37a may mediate the high selectivity of the TSHR. Therefore, follicle-stimulating hormone (FSH) and luteinizing hormone (LH) receptors are unaffected by this compound. S37a not only inhibited the activation of TSHR by TSH itself but also inhibited the activation of monoclonal TSAb M22, KSAb1, and the allosteric small-molecule agonist C2. S37a also inhibited cAMP formation in oligoclonal TSAb used for GO treatment. S37a is a novel and highly selective TSHR inhibitor. This inhibitor has the potential for development for the treatment of GO.

## 3 Thyroid hormone receptor

**FIGURE 3:**
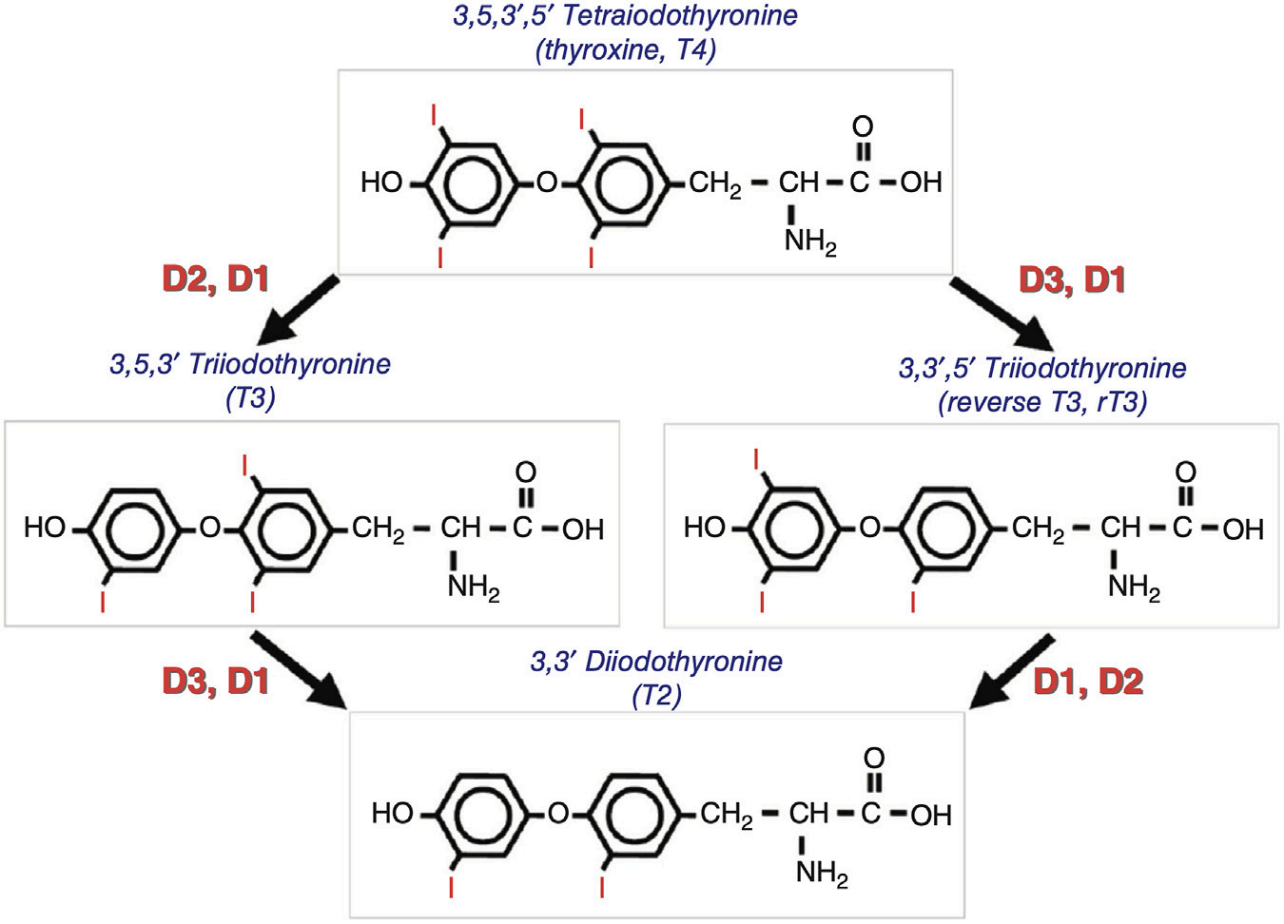
Schematic representation of the deiodinase-mediated activation or inactivation of thyroxine and triiodothyronine^[3]^.

TH are synthesised in the thyroid gland and contains triiodothyronine (T3) and thyroxine (T4). T4 is the predominant form of TH. T3 is the active form in tissues in which T4 undergoes deiodination. TH signaling is strictly regulated by iodothyronine deiodinase (Dio) activity, which both preserves the circulating levels of the biologically active triiodothyronine (T3) and regulates TH homeostasis at the local level, in a cell- and time-dependent manner. (Moran et al., 2022). Dio includes three types, among which Dio1 and Dio2 mainly convert T4 into more active T3; Dio3 can inactivate T3 and T4 to T2 and rT3. (Ortiga-Carvalho et al., 2016) (Figure 3). An imbalance in TH homeostasis leads to susceptibility to obesity and metabolic disorders. T3 binds to TR; regulates cell metabolism, growth, and development; and plays a role in a series of physiological and pathological processes. (Yen et al., 2006). The pharmacological status of TR proteins is closely related to physiological conditions.

TR is structurally comprised of three parts: the N-terminal region, the DNA-binding region (DBD), and the ligand-binding region (LBD). Each gene had two subtypes. TRα1 and TRα2 are produced by differential splicing of precursor mRNA, and the difference between them is mainly located at the carboxyl terminal. TRβ1 and TRβ2were obtained by differential expression of promoters, and these two subtypes were only different at the amino terminal. Different organisations and developmental stages produce different expression patterns. (Banta et al., 2018). TRα2 is mainly located in the pituitary and CNS. Unlike other TR, this subtype does not bind to ligand molecules and mainly acts as transcriptional inhibitors. TRα1 is also more widely distributed, with studies showing that most TRα1 have effects on the heart. In addition, TR of subtype β is mainly located in the liver and other tissues. (Lazcano et al., 2019). TH regulates gene expression primarily by binding to TR. The TR, with an unbound ligand molecule, consists of 12 β-helices and a short β-angle. Such a structure predisposes TR proteins to bind to corepressors by forming hydrophobic interactions, which in turn repress gene transcription. (Webb et al., 2000).

TR regulators can be classified as agonists and antagonists. When the TR agonist molecule is present, it occupies the ligand-binding pocket on TR, thus changing the conformation of the receptor. The difference in TR between the bound agonist molecule and the unbound ligand was mainly located in the carbon terminal helix. TR without binding agonist molecules, helix H11 and helix H10, are vertical and face the ligand-binding pocket. After agonist molecules bind to the receptor, helices H11 and H10 are connected in series, whereas helix H12 is reset in the ligand-binding pocket. This further leads to the dissociation of co-inhibitors and the recruitment of co-activators. Further activation of gene transcription using the above methods. Some HR antagonists are similar to TH and can replace spiral H12. Other HR antagonists can bind to the TH-binding pocket, but fail to form a hydrogen bond network. A hydrogen-bonded network is necessary for spiral H12 stacking. When bound with an antagonist, HR helix H12 rotated 120°. (Webb et al., 2002). Therefore, it faces the amino terminus of the ligand-binding region and inhibits gene transcription. Recently, several TR regulators have been synthesised (Table 2).

**TABLE 2:** Some regulators of thyroid hormone receptor.

Year	Researchers	Name	Binding site	Action	Ref.
2002	Lim et al.	HN-3	TRβ1	Antagonist	Lim et al. (2002)
2003	Schapira et al.	1–850	TRα1and TRβ1	Antagonist	Schapira et al. (2003)
2007	Erion et al.	VK2809 (MB08711)	TRβ	Agonist	Erion et al. (2007)
2007	Estébanez-Perpiñá et al.	DHPPA	TRβ1	Antagonist	Estébanez-Perpiñá et al. (2007)
2008	Berkenstam et al.	KB2115 (Eprotirome)	TRβ1	Agonist	Berkenstam et al. (2008)
2010	Scanlan et al.	GC-1	TRβ1	Agonist	Scanlan (2010)
		(Sobetirome)			
2011	Hwang et al.	MLS389544	TRβ1	Antagonist	Hwang et al. (2011)
2014	Ogungbe et al.	Lignans	TRβ	Antagonist	Ogungbe et al. (2014)
2014	Kelly et al.	MGL-3196 (Resmetirom)	TRβ	Agonist	Kelly et al. (2014)
2019	Hartley et al.	Sob-AM2	TRβ	Agonist	Hartley et al. (2019)
2020	Perra et al.	IS25	TRβ	Agonist	Perra et al. (2020)
2020	Perra et al.	TG68	TRβ	Agonist	Perra et al. (2020)
2021	Panda et al.	Syringic acid	TRβ	Agonist	Panda et al. (2021)

### 3.1 GC-1

GC-1 (sobetirome) is a TRβ1selective agonist. TH are easier to synthesise and modify than endogenous TH. The specific binding of GC-1 to TRβ1 in liver is 6 times that of TRα1. Therefore, GC-1 mainly plays a lipid regulatory role in the liver. GC-1 decomposes and clears cholesterol by upregulating low-density lipoprotein receptors (LDLR) in the liver and stimulating the reverse transport of cholesterol to form bile acids. In a Phase I clinical trial, GC-1 reduced low-density lipoprotein cholesterol (LDL-C) by 41% after 2 weeks of treatment and was well tolerated in patients with normal thyroid function. (Tancevski et al., 2011). GC-1 can also increase the expression of SR-B1 in the liver, thereby promoting the reverse transport of cholesterol to form bile acid to clear cholesterol. The main mechanism is not through the transcription of SR-B1 mRNA but by increasing the translation of SR-B1 protein to play a role in regulate lipids. In addition, GC-1 can enhance CYP7A1 activity and induce the expression of the adenosine triphosphate-binding transporter (ABCG5/ABCG8) in the liver to promote bile acid decomposition of cholesterol. At the same time, it stimulates the oxidation of fatty acids in the mitochondria, reducing or even eliminating TG accumulation in the liver. (Lin et al., 2012). Moreover, GC-1 can also promotes energy consumption and mitochondrial oxidation. (Scanlan, 2010). GC-1 cholesterol lowering therapy may be a good choice for the treatment of familial homozygous hypercholesterolaemia lacking LDLR.

### 3.2 KB2115

KB2115 (eprotirome) is a TRβ1 selective agonist. The lipid-regulating effects of KB2115 play a role in increasing cholesterol excretion. In a clinical study of patients with hypercholesterolaemia and a high body mass index, after 2 weeks of treatment with KB2115, the levels of serum total TC, LDL-C, and ApoB decreased, and no cardiac complications such as arrhythmia occurred. In the Phase I and Phase II clinical studies, KB2115 was used as a single drug to treat hypercholesterolaemia, and its affinity with TRβ1 was 22 times that of TRα1. (Berkenstam et al., 2008). Ladenson et al (Ladenson et al., 2010c) conducted a study in which patients with dyslipidaemia after statin administration were randomised into four groups that were administered three different doses of KB2115 and continued statin lipid-modifying drugs. After 12 weeks of treatment, plasma LDL-C levels in the high-dose, middle-dose, low-dose, and placebo groups decreased from baseline by 32%, 28%, 22%, and 7%, respectively, in a dose-dependent manner. Among these, the LDL-C, TG, ApoB, and lipoprotein(a) levels decreased significantly in the high-dose group. It was not associated with cardiac, skeletal, or pituitary disorders. Meanwhile, both total thyroxine (TT4) and free thyroxine (FT4) levels decreased in the KB2115 group, whereas total triiodothyronine (TT3) and free triiodothyronine (FT3) levels remained unchanged. This suggested that KB2115 and endogenous TH feed together on the HPT axis to regulate the synthesis and secretion.

### 3.3 NH-3 and lignans

NH-3 is a first-generation antagonist molecule acting on TR, and research has shown that this compound can inhibit the function of TH in cell culture and whole animal experiments. (Lim et al., 2002; Zucchi, 2020). When bound with TR, NH-3 competitively inhibits the recruitment of TRE-containing reporter genes and p1 (Caddeo et al., 2021) co-activating proteins. NH-3 can also significantly induce differences in TR conformational changes, resulting in incorrect folding of helix 12. NH-3 also antagonises TH-induced changes and upregulates the TH-regulated gene collagenase 3. NH-3 antagonises TR in animal models, and thus represents a TR antagonist that can be used *in vivo*. Lignans are important bioactive polyphenolic compounds in the diet. Eating foods rich in lignans has a positive impact on health. Lignans (−) arctigenin and (+) pinoresinol is the antagonist molecule of TRβ. The structure of the compound matched that of the TRβ binding pocket, and its binding activity was high. Moreover, it reacts with the amino acid residues Arg 282, His435, Ala279 and Met 313 in the binding pocket, thus playing an antagonistic role. (Ogungbe et al., 2014).

## 4 Thyrotropin releasing hormone receptor

TRH, secreted by the hypothalamus, is the strongest regulator of the HPT axis. TRH binds to the surface-specific receptor of TRH in the anterior pituitary, which not only stimulates the synthesis and secretion of TSH in the adenohypophysis, but also participates in the regulation of respiratory, cardiovascular, and other physiological functions as a neurotransmitter or modulator. TRH regulates prolactin secretion in the anterior pituitary gland. (Joseph-Bravo et al., 2015).TRH is used to treat brain and spinal cord injuries as well as CNS diseases, including epilepsy, schizophrenia, spinal cord trauma, Alzheimer’s disease (AD), Parkinson’s disease (PD), and depression. (Khomane et al., 2011b).

TRHR is an important member of the rhodopsin-like G protein-coupled receptor family. Intracellular signal transduction is mainly mediated by coupling with the Gq/11 protein. The combination of TRH and its receptor leads to the activation of phospholipase C, which stimulates the hydrolysis of PIP2 to form InsP3 and DAG. These secondary messengers stimulate an increase in intracellular calcium ions and activate protein kinase C (PKC). (Hsieh and Martin, 1992). TRH activation also stimulates calcium/calmodulin-dependent protein kinase and mitogen-activated protein kinase (MAPK) activities. (Cui et al., 1994). TRH has also been shown to activate Gi2, Gi3 or Gs proteins, but not adenylate cyclase. (Gershengorn and Osman, 1996). It has been proved that some transcription factors induce gene transcription under the regulation.

TRH-R1, the first TRHR discovered in humans, was cloned from a cDNA library of mouse pituitary tumours. TRH-R2 and TRH-R3 were subsequently identified. (Li et al., 2020).The pituitary gland is the main site of TRH. TRHR is mainly distributed in the TSH cells of the pituitary gland, as well as on the surface of PRL cells, the CNS, and its peripheral tissues, including the testis, stomach, small intestine, colon, adrenal medulla, and pancreas. In the human body, only human TRH-R1 has been reported, lacking TRH-R2. In some mammals, TRH-R2 is responsible for neuropharmacological effects, whereas TRH-R1 is responsible for endocrine activity. (Charli et al., 2020).Therefore, the related TRHR regulators also produce biological effects, mainly by acting on these two receptors.

Several TRHR regulators have been identified, including azetirelin, NP-647, DN-1417, and JTP-2942. (Khomane et al., 2011c). NP-647 selectively binds to the TRH-R2 receptor and is an effective antiepileptic drug with negligible endocrine side effects. However, the half-life of NP-647 is short. Therefore, it is important to identify TRHR regulators that can be widely used in clinical practice. Currently, research on TRHR regulators mainly focuses on two drugs: taltirelin and rotatirelin.

### 4.1 Taltirelin

Taltirelin (TA-0910) is an orally administered TRHR agonist. Compared to TRH, taltirelin stimulates the central nervous system more strongly. Taltirelin is approved for the treatment of spinocerebellar degeneration (SCD). Therefore, it is extremely promising to study other possible applications of TRH. Choi et al. (Choi et al., 2015) study that the application of taltirelin can change depressive behaviour through TRHR in the basolateral amygdala (BLA). Zheng et al. (Zheng et al., 2018) showed that taltirelin, both *in vitro* and *in vivo*, accompanied by elevated p-ERK1/2 levels, mediates resistance to apoptosis. Taltirelin improves motor function in animal models of mitochondrial inhibitor-induced neuronal injury. Simultaneously, TSH and TH levels in the serum of mice were measured. When the taltirelin dose exceeded 0.2 mg/kg, total T4 increased significantly, whereas FT4 and FT3 did not change. Thus, the results suggest that patients’ thyroid function should be thoroughly assessed and adjusted accordingly before administering taltirelin when treating neurological disorders. Recent research has shown that taltirelin is a stable upper respiratory tract stimulant with wake-up characteristics. These characteristics have a potential beneficial correlation with some respiratory diseases but have nothing to do with other respiratory diseases. (Liu et al., 1985).

### 4.2 Rovatirelin

Rovatirelin ([1-[-[(4S,5S)-(5-methyl-2-oxo oxazolidin-4-yl) carbonyl]-3-(thiazol-4-yl)-L-alanyl]-(2R)-2- methylpyrrolidine) is a TRHR modulator that mimics the TRH. Ijiro et al. (Ijiro et al., 2015) investigated the electrophysiological and pharmacological effects of rovatirelin on the central noradrenergic system and compared their results with those of taltirelin. Rovatirelin binds to human TRHR with higher affinity than talirelin. Rovatirelin increases the spontaneous firing of action potentials of acutely dissociated noradrenergic neurones in the rat locus coeruleus (LC). The facilitating effect of rovatirelin on the firing rate of LC neurones was inhibited by the TRHR antagonist, chlorodiazepoxide. These results suggest that rovatirelin exerts CNS-mediated effects through the CNS noradrenergic system and is more potent than taltirelin. Further, Ijiro et al. (Ijiro et al., 2020) found that in spinocerebellar ataxia (SCA) model animals, rovatirelin, which activates the cerebellum and other parts of the central nervous system to improve motor function, is more effective than taltirelin. Rovatirelin also improve motor dysfunction in a cytarabine-induced rat model of spinocerebellar degeneration (SCD) via acetylcholine and dopamine neurotransmitters (Ijiro et al., 2022). It follows that rovatirelin may have orally effective therapeutic potential for patients with SCD and SCA (Table 3).

**TABLE 3:** Characteristics of some receptor modulators in this review.

Modulator	Receptor	Specificity	Synthetic or not	Administration route	Function
M22	TSHR	LRD of TSHR	N	Intramuscular or intravenous	Activate TSHR
K1-18	TSHR	LRD of TSHR	N	Intramuscular or intravenous	Activate TSHR
5C9	TSHR	LRD of TSHR	N	Intramuscular or intravenous	Inhibition of TSHR activation
K1-70	TSHR	LRD of TSHR	N	Intramuscular or intravenous	Inhibition of TSHR activation
NCGC00161870	TSHR	TSHR	Y	Oral	Activate TSHR
MS437	TSHR	TMD of TSHR	Y	Intraperitoneal injection	Increase the release of TH
MS438	TSHR	TMD of TSHR	Y	Intraperitoneal injection	Increase the release of TH
NCGC00229600	TSHR	TSHR	Y	Oral	Inhibition of TSHR activation
S37a	TSHR	Ectodomain/TMD of TSHR	Y	Oral	TSHR antagonist
GC-1	TR	TRβ	Y	Intraperitoneal injection	TRβ1 agonist
KB2115	TR	TRβ	Y	Oral	TRβ1 agonist
NH-3	TR	TR	Y	NA	TR antagonist
Lignans	TR	TRβ	Y	NA	TRβ antagonist
Taltirelin	TRHR	TRHR	Y	Oral	TRH analog
Rovatirelin	TRHR	TRHR	Y	Oral	TRH analog

TMD, transmembrane domain; LRD, leucine-rich domain; Y, yes; N, no; NA, not available.

## 5 Conclusion

Several congenital and acquired factors affect thyroid development and function. The core is the effect of the hypothalamus-pituitary-thyroid (HPT) axis. As the main pathway of endocrine regulation in humans, the HPT axis is involved in the development of several systemic diseases, including digestive, cardiovascular, and CNS diseases, by regulating the production and secretion of TSH, TH, and TRH. In a healthy person, the HPT axis must be in a balanced state of regulation and a feedback system. TSH, TH, and TRH exert their biological effects by binding to the related receptors. The three receptors, TSHR, TR, and TRHR, are important in human diseases.

Through receptor modulators, the binding of TSHR, TR, and TRHR to their corresponding ligands can be altered, stimulating or inhibiting downstream signalling pathways and altering disease states. For example, a receptor activator acts as a ligand mimic to facilitate subsequent reactions by binding to the receptor. By contrast, downstream responses can be inhibited by a receptor inhibitor after binding to the corresponding receptor. Although many HPT axis receptor modulators have been identified, few drugs can be used in clinical practice. However, the use of receptor modulators is accompanied by adverse effects. In recent years, small-molecule ligand drugs have gradually become a hotspot in the development of receptor modulators. However, most small-molecule ligand drugs remain in the experimental stages of development. It takes some time to enter the clinic. Therefore, the study of novel modulators is crucial for disease treatment. Therefore, further development of receptor modulators of the HPT axis remains a great challenge and is a goal of our endeavours.

Driven by the development of modern Genomics, Proteomics, and bioinformatics, the number of receptor modulators has increased rapidly, bringing great opportunities and challenges to drug design and new drug research and development based on receptor structure. For example, AITD, a TSHR-related disease, can be a hot disease field for precise treatment and will be an important direction for the development of receptor regulators. Although the clinical efficacy of receptor modulators for HPT axis disease is still not ideal, researchers are exploring this further. We have reasons to believe that with the continuous discovery of new receptor modulators and the increasingly mature application of new technologies, safer and more effective receptor modulators and new drugs will continue to emerge, shedding light on the treatment of HPT axis diseases and benefiting humanity. Our team has mainly studied the interaction between proteins through molecular dynamics simulation and experimental techniques in recent years and has achieved some results in these works. (Guan et al., 2018; Guan et al., 2020; Fan et al., 2023). In the future, we will conduct in-depth research on the modulators of HPT axis receptors. We hope that our research will provide new insights into the diagnosis and treatment of HPT axis-related diseases.
