# Supplementary material for: Receptor modulators associated with the hypothalamus -pituitary-thyroid axis
Source: Front Pharmacol. 2023 Dec 4;14:1291856. doi: 10.3389/fphar.2023.1291856 (PMC10725963; doi:10.3389/fphar.2023.1291856)
Supplement: Supplementary file 1 [file Table1.DOCX]

TABLE 1 Some small molecule ligands of thyrotropin receptor

| **Year** | **Researchers** | **Name** | **2D structure** | **3D status** | **Action** |
| --- | --- | --- | --- | --- | --- |
| 2006 | Jaschke H et al. ^41^ | Org41841 | 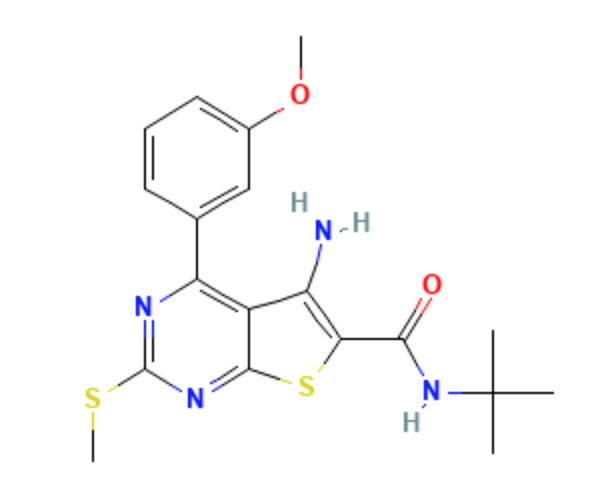 | 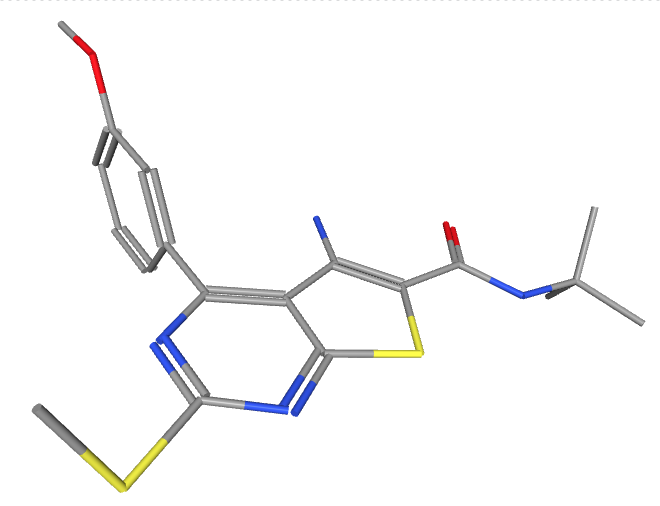 | Agonist |
| 2009 | Neumann S et al. ^35^ | NCGC00161870 （C2/ML-109) | 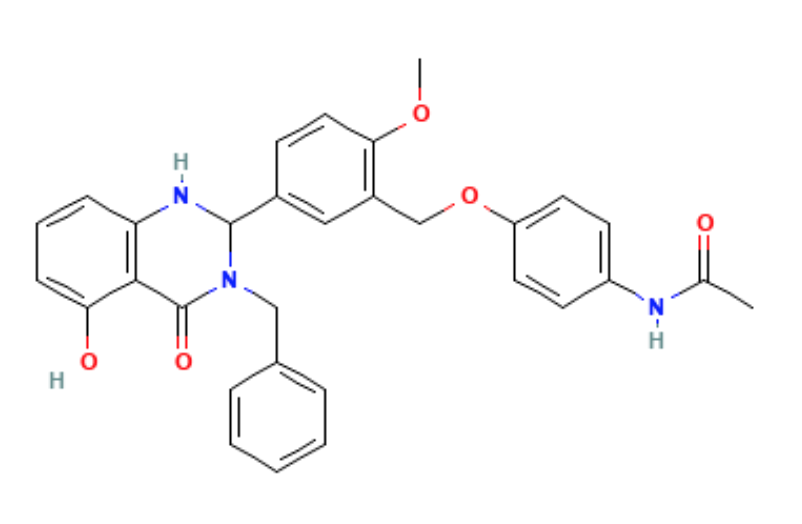 | 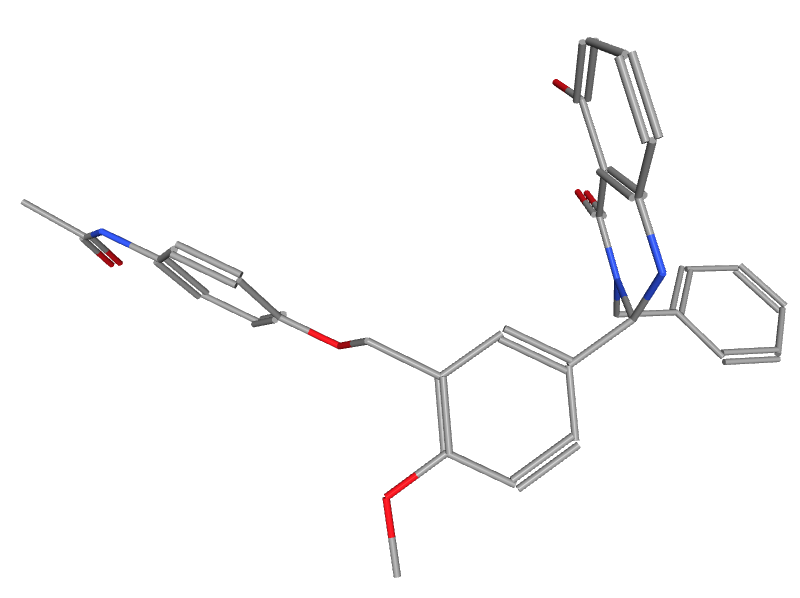 | Agonist |
| 2010 | Neumann S et al. ^42^ | NCGC00161856 | 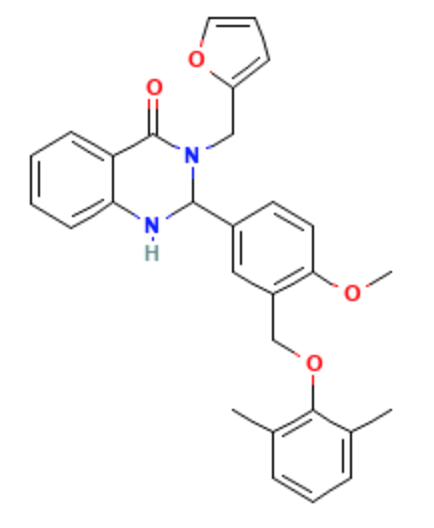 | 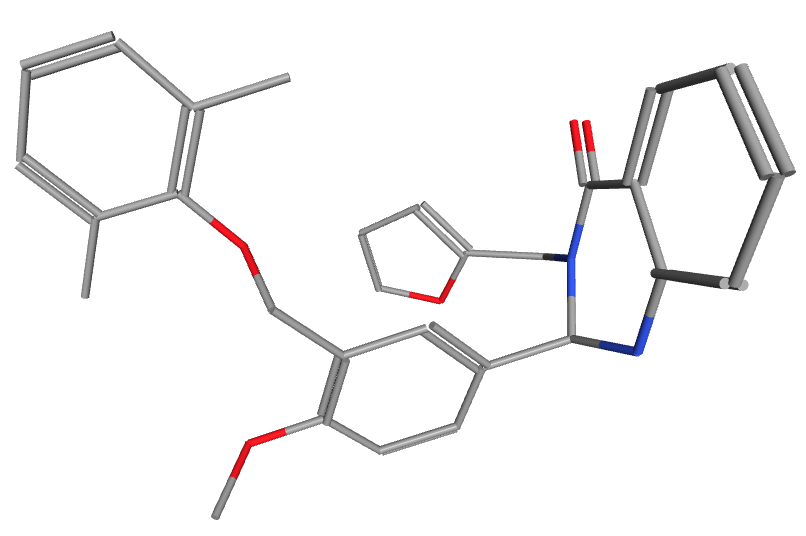 | Antagonist |
| 2011 | Neumann S et al. ^38^ | NCGC00229600 | 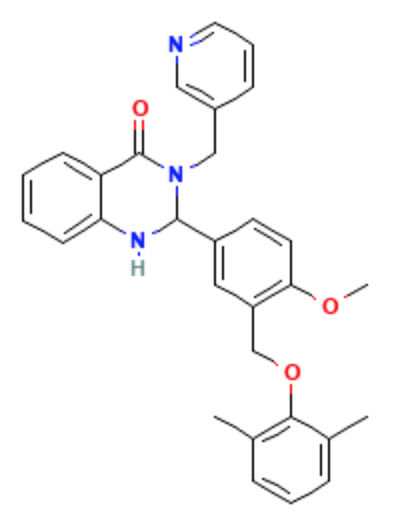 | 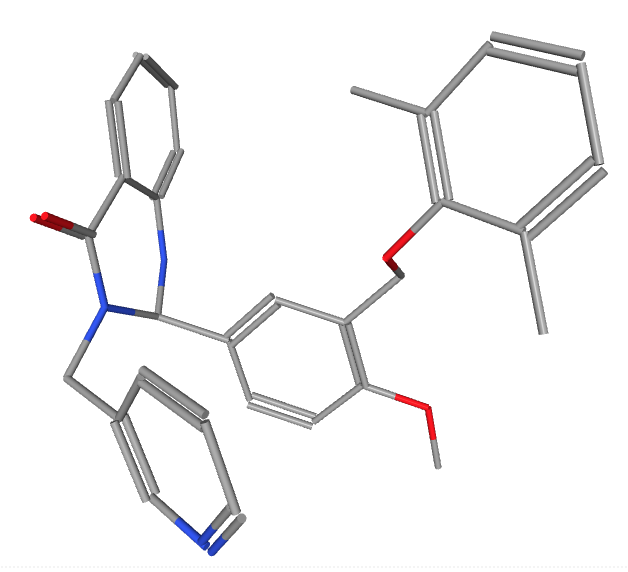 | Inverse agonist |
| 2014 | Neumann S et al. ^43^ | NCGC00242364 (ML-224/ANTAG-3) | 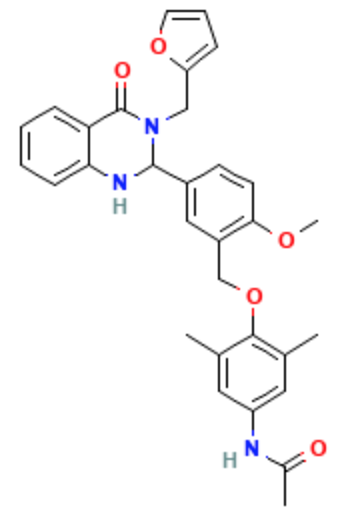 | 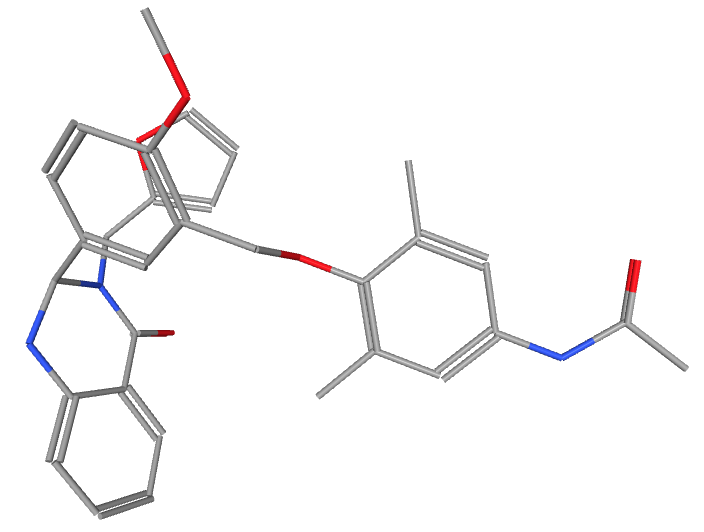 | Antagonist |
| 2015 | Latif R et al. ^37^ | MS437 | 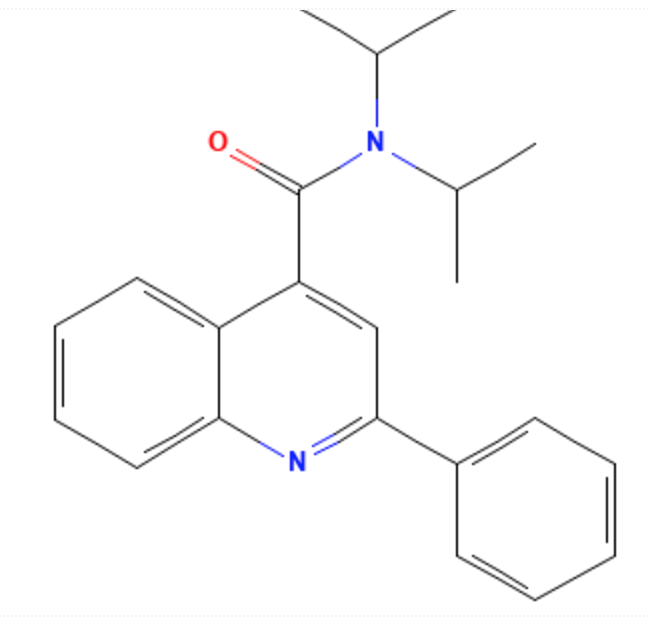 | 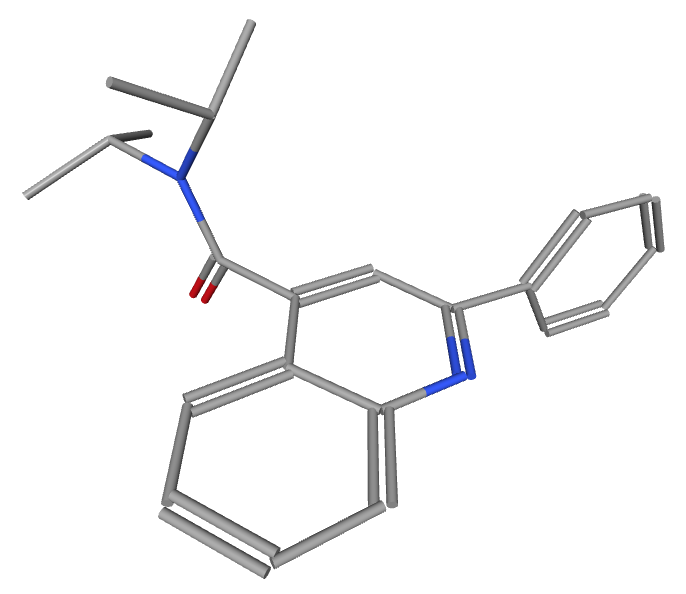 | Agonist |
| 2015 | Latif R et al. ^37^ | MS438 | 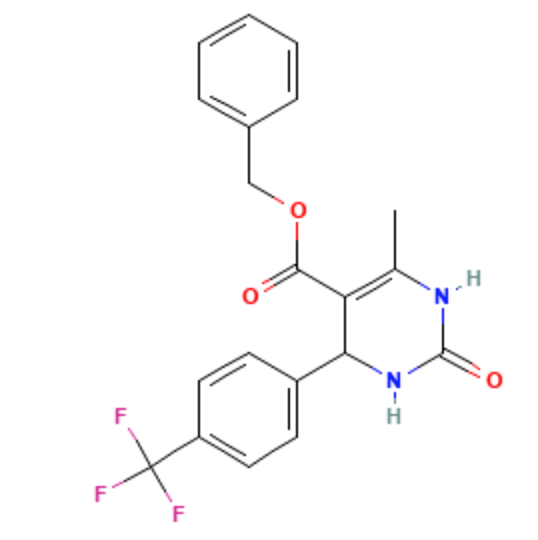 | 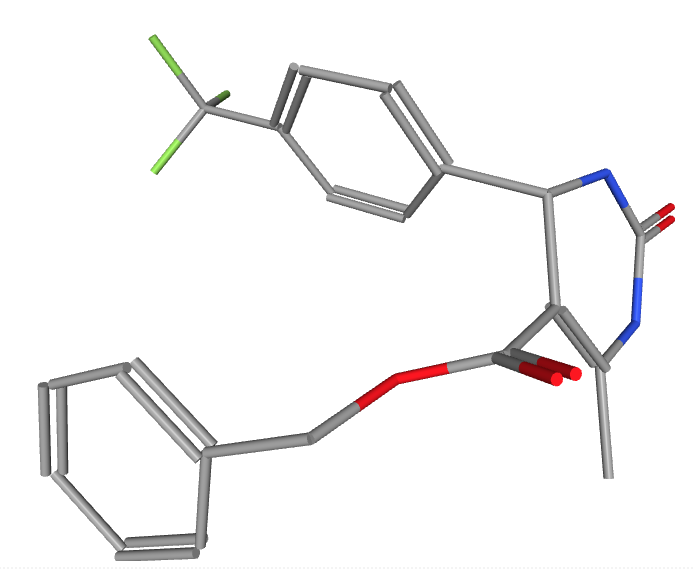 | Agonist |
| 2019 | Marcinkowski P  et al. ^40^ | S37a | 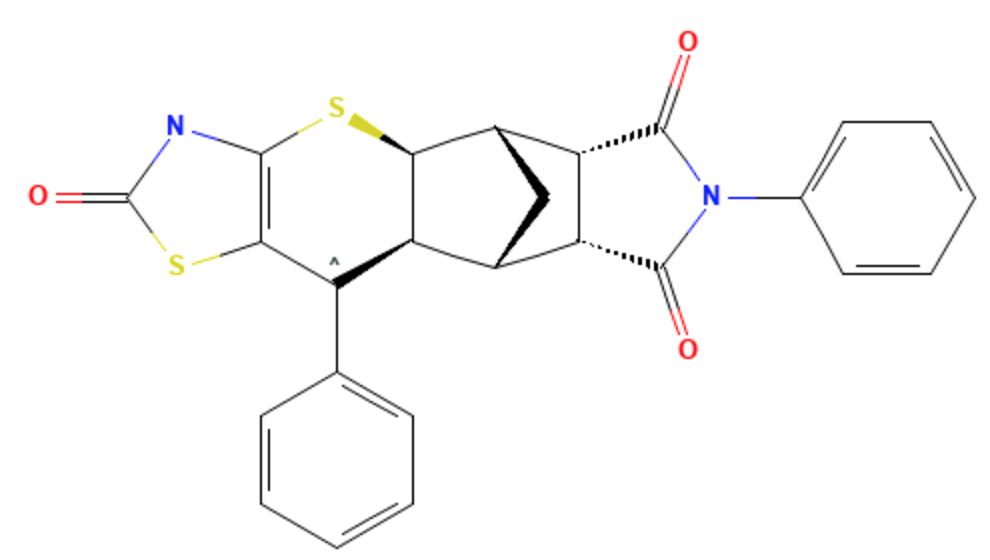 | 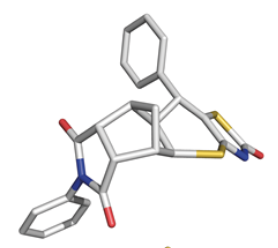 | Antagonist |
| 2020 | Latif R et al. ^44^ | MSq1 | 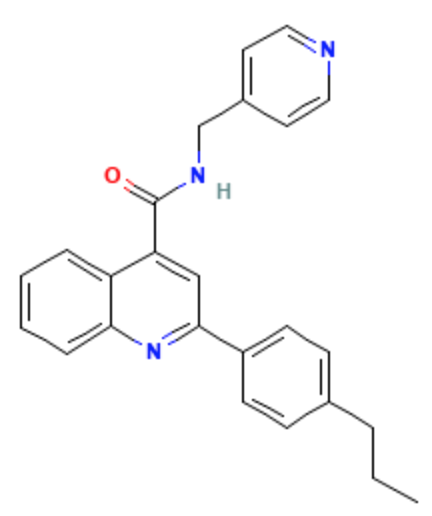 | 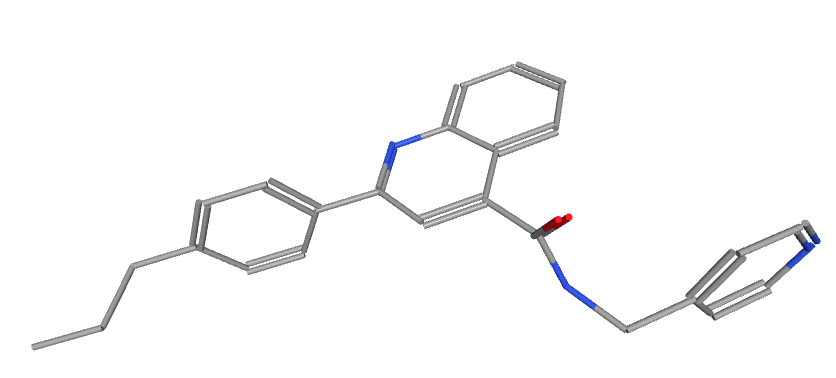 | Agonist |
