# Supplementary material for: Receptor modulators associated with the hypothalamus -pituitary-thyroid axis
Source: Front Pharmacol. 2023 Dec 4;14:1291856. doi: 10.3389/fphar.2023.1291856 (PMC10725963; doi:10.3389/fphar.2023.1291856)
Supplement: Supplementary file 2 [file Table2.DOCX]

TABLE2 Some regulators of thyroid hormone receptor

| **Year** | **Researchers** | **Name** | **Binding site** | **Action** | **Ref.** |
| --- | --- | --- | --- | --- | --- |
| 2002 | Lim et al. | HN-3 | TRβ1 | Antagonist | 56 |
| 2003 | Schapira et al. | 1-850 | TRα1and TRβ1 | Antagonist | 61 |
| 2007 | Erion et al. | VK2809  (MB08711) | TRβ | Agonist | 62 |
| 2007 | Estébanez-Perpiñá et al. | DHPPA | TRβ1 | Antagonist | 63 |
| 2008 | Berkenstam et al. | KB2115  (Eprotirome) | TRβ1 | Agonist | 54 |
| 2010 | Scanlan et al. | GC-1  （Sobetirome） | TRβ1 | Agonist | 53 |
| 2011 | Hwang et al. | MLS389544 | TRβ1 | Antagonist | 64 |
| 2014 | Ogungbe et al. | Lignans | TRβ | Antagonist | 58 |
| 2014 | Kelly et al. | MGL-3196  (Resmetirom) | TRβ | Agonist | 65 |
| 2019 | Hartley et al. | Sob-AM2 | TRβ | Agonist | 66 |
| 2020 | Perra et al. | IS25 | TRβ | Agonist | 59 |
| 2020 | Perra et al. | TG68 | TRβ | Agonist | 59 |
| 2021 | Panda et al. | Syringic acid | TRβ | Agonist | 67 |
