# Supplementary material for: Receptor modulators associated with the hypothalamus -pituitary-thyroid axis
Source: Front Pharmacol. 2023 Dec 4;14:1291856. doi: 10.3389/fphar.2023.1291856 (PMC10725963; doi:10.3389/fphar.2023.1291856)
Supplement: Supplementary file 3 [file Table3.DOCX]

| **TABLE 3 Characteristics of some receptor modulators in this review.** | | | | | |
| --- | --- | --- | --- | --- | --- |
| **Modulator** | **Receptor** | **Specificity** | **Synthetic or not** | **Administration route** | **Function** |
| M22 | TSHR | LRD of TSHR | N | Intramuscular or intravenous | Activate TSHR |
| K1-18 | TSHR | LRD of TSHR | N | Intramuscular or intravenous | Activate TSHR |
| 5C9 | TSHR | LRD of TSHR | N | Intramuscular or intravenous | Inhibition of TSHR activation |
| K1-70 | TSHR | LRD of TSHR | N | Intramuscular or intravenous | Inhibition of TSHR activation |
| NCGC00161870 | TSHR | TSHR | Y | Oral | Activate TSHR |
| MS437 | TSHR | TMD of TSHR | Y | Intraperitoneal injection | Increase the release of TH |
| MS438 | TSHR | TMD of TSHR | Y | Intraperitoneal injection | Increase the release of TH |
| NCGC00229600 | TSHR | TSHR | Y | Oral | Inhibition of TSHR activation |
| S37a | TSHR | Ectodomain/TMD of TSHR | Y | Oral | TSHR antagonist |
| GC-1 | TR | TRβ | Y | Intraperitoneal injection | TRβ1 agonist |
| KB2115 | TR | TRβ | Y | Oral | TRβ1 agonist |
| NH-3 | TR | TR | Y | NA | TR antagonist |
| Lignans | TR | TRβ | Y | NA | TRβ antagonist |
| Taltirelin | TRHR | TRHR | Y | Oral | TRH analog |
| Rovatirelin | TRHR | TRHR | Y | Oral | TRH analog |
| TMD, transmembrane domain; LRD, leucine-rich domain; Y, yes; N, no; NA, not available. | | | | | |
